# Predicting Acute Myocardial Infarction During Acute Ischemic Stroke: Need for a Screening Tool Beyond Biomarkers∗

**DOI:** 10.1016/j.jacasi.2022.10.003

**Published:** 2022-11-29

**Authors:** Sung-Ho Ahn, Sun U. Kwon

**Affiliations:** a Department of Neurology, Division of Biostatistics, Research Institute for Convergence of Biomedical Science and Technology, Pusan National University, Yangsan Hospital, Pusan National University School of Medicine, Busan, Korea; b Department of Neurology, Asan Medical Center, University of Ulsan, College of Medicine, Seoul, Korea

**Keywords:** acute ischemic stroke, acute myocardial infarction, prediction model

**Figure:**
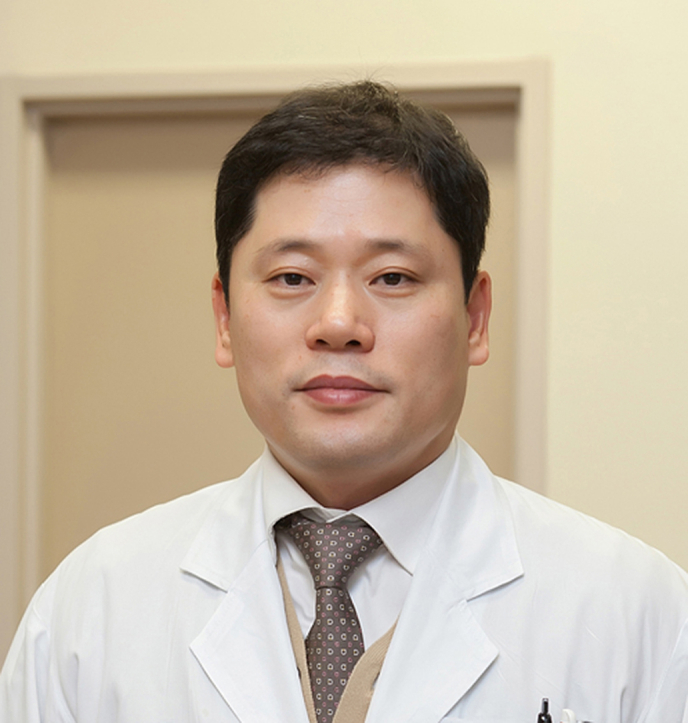

Ischemic stroke and ischemic heart disease (IHD) share common risk factors and pathological mechanisms.^1^ Consequently, a higher proportion of patients with ischemic stroke have concomitant coronary artery disease and thus an increased risk of developing ischemic stroke and death.^2^ Since the publication of the 2007 guidelines for the management of acute ischemic stroke (AIS), all patients with AIS are recommended to undergo cardiac enzyme testing—particularly cardiac troponin, a highly sensitive and specific biomarker of myocardial damage^3^—and 12-lead electrocardiography (ECG).^4^ However, identifying IHD, especially acute myocardial infarction (AMI), remains a diagnostic challenge in patients with AIS. The difficulty is that these patients are often elderly, and the incidence of concomitant cardiac pathology is often high.^5^ Besides, identifying AMI symptoms in patients with AIS is challenging because these patients often have difficulties communicating because of neurological deficits or cognitive dysfunctions induced by stroke.

In this issue of *JACC: Asia*, Chen et al^6^ from China present the results of a newly developed simple risk stratification score (ie, the CTRAN [Coronary Heart Disease, Malignant Tumor, Renal Insufficiency, Age, National Institutes of Health Stroke Scale] score), based on bedside accessible information, which could be used effectively to identify patients at high risk of in-hospital AMI in a population of Asian patients with AIS. Among a total of 96,367 selected patients registered in the China nationwide multicenter registry from 2016 to 2020, 392 patients (0.41%) suffered from in-hospital AMI, and 49 of the 392 (12.5%) were diagnosed with AMI before discharge. Chen et al^6^split the pooled cohort into a derivation cohort (n = 65,189, from 70 centers) and a validation cohort (n = 31,178, from 22 centers). Based on the results from the derivation cohort, they proposed the CTRAN score. This scoring system was derived by calculating performance using the area under the curve (AUC) analysis (AUC = 0.777; 95% CI: 0.746-0.808). The proposed score also had a good performance in the validation cohort with an AUC of 0.753 (95% CI: 0.714-0.793); besides, it was still consistent in the subgroups analysis, including analysis based on ischemic lesion location, which considered a possible bias of National Institutes of Health Stroke Scale score (according to the location). The CTRAN score could be a good tool for the clinical identification of patients at high risk of in-hospital AMI after AIS.

Regarding the biological plausibility of using the CTRAN score to predict AMI, a history of coronary heart disease has long been known to be the most potent predictive factor of future AMI. Malignant tumors increase the risk of cancer-related hypercoagulability and chemotherapy-induced cardiomyopathy, which may be the reason for the increased risk of AMI. In addition, the burden of cardiovascular diseases, including AMI, is known to increase gradually with age. Finally, the brain-heart axis concept, involving autonomic deregulation and catecholamine surge, might play a dominant role in the aggravation of the underlying coronary artery/myocardial injury and the occurrence of AMI (type II MI). Thus, Chen et al^6^ concluded that the CTRAN score is of clinical significance because it can be used as a predictive tool for AMI in a practical field with the following strengths: 1) it was derived from a large multicentric database comprising a broad spectrum of Chinese patients with AIS; and 2) the score was validated in an independent external cohort and showed good discrimination. However, Chen et al^6^ also acknowledged some limitations, such as the lack of follow-up of AMI events, baseline troponin data, and generalizability across races.

Traditionally, sequential assessment of serum cardiac troponin level and ischemic ECG changes have been regarded as the gold standard for diagnosing AMI, because cardiac troponins are sensitive and specific biomarkers of cardiac injury. Furthermore, troponin level elevation has also been frequently observed^7^ and is associated with an increased risk of death and/or disability in patients with AIS.^8^ However, analyzing troponin elevation in patients primarily presenting with AIS is far more complex. First, concomitant IHD can be an important cause of troponin elevation in patients with AIS. Second, other physiological conditions such as atrial fibrillation, congestive heart failure, ventricular hypertrophy, chronic kidney disease, or active cancer could be significant sources of troponin elevation. Finally, neurally mediated cardiac injury via autonomic dysfunction after stroke based on the brain-heart axis concept is another factor leading to troponin elevation.^9^ Therefore, the stroke itself has been classified in the category of multifactorial or indeterminate causes of myocardial injury^3^ or systemic conditions, for other reasons of myocardial injury,^10^ according to the recent publication of the “Fourth Universal Definition of Myocardial Infarction.” In this regard, defining actual AMI in patients with AIS remains a challenge. As noted, the accurate diagnosis of AMI, especially during AIS, is quite tricky for acquiring AMI symptoms caused by neurological deficits. Furthermore, conversely, even when attempting to accurately diagnose AMI using cardiac biomarkers (including serum troponin) and ECG, there is also a risk of overestimating the incidence of AMI because an abrupt surge of troponin can occur in patients with AIS, via a neurally mediated cardiac injury similar to AMI. For this reason, this extensive database study, based on a nationwide registry, is limited in that it could both under- and overestimate AMI and be biased, particularly regarding the diagnosis of AMI.

However, despite these limitations, developing a readily valuable screening tool such as the CTRAN score may be helpful to sort out patients who have AIS and are considered high risk, who could be potential candidates for intensive neurocardio monitoring for the following reasons. At first, although the current guideline recommends for routine measurement of serum cardiac enzyme with a 12-lead ECG test,^4^ routine measurements of these biomarkers are not always available throughout all medical centers, considering the lack of infrastructure for managing 24-hour laboratory facilities or increasing medical costs for these tests. In this sense, the CTRAN score can be a cost-free screening tool based on bedside accessible information to identify patients at high risk of in-hospital AMI in the setting of the AIS. In the practical field, the CTRAN score can be used as an indicator to perform a sequential test, including the measurement of serum cardiac enzyme with an ECG test, especially for patients at high risk who have a high CTRAN score. Furthermore, the CTRAN score also can be used to select candidates for repeated measurement of cardiac biomarkers among patients with AIS. Until now, the current guidelines for managing AIS recommend repeated measures in some cases where developing silent ischemia or paroxysmal arrhythmia is suspected. However, likewise diagnosing AMI based on a rise and/or fall of troponin levels and assessment of dynamic changes of troponin value is essential for the differential diagnosis of the cause of cardiomyocyte damage to distinguish acute from chronic elevations. A recent prospective cohort study also confirms the practical value of repeated measurement of serum cardiac enzyme with a combination of serial ECGs, which improve the detection and tracking performance of troponin value and their risk stratification power.^8^ Therefore, the CTRAN scoring system has a lot of potential to be used as a screening tool for the early detection of subjects at high risk who need intensive neurocardiac monitoring regarding in-hospital AMI. Finally, the CTRAN score will ensure early and accurate detection of in-hospital AMI and, in turn, improve patient outcomes. We look forward to seeing the next step of this study to enhance the clinical utility of cardiac monitoring in patients with AIS for the prevention, early detection, and appropriate management of IHD, including AMI.

## Funding Support and Author Disclosures

The authors have reported that they have no relationships relevant to the contents of this paper to disclose.
